# Ultrasonography of lateral elbow pain through a weighted varus flexion position contributes to detect minor instabilities

**DOI:** 10.1016/j.jseint.2024.11.003

**Published:** 2024-11-28

**Authors:** Aurelien Traverso, Valeria Vismara, Simone Cassin, Andrea Zagarella, Pietro Randelli, Paolo Arrigoni

**Affiliations:** a Department of Orthopaedics and Traumatology, Centre hospitalier universitaire vaudois (CHUV), Lausanne, Switzerland; b Clinica Ortopedica, Azienda Socio Sanitaria Territoriale Centro Specialistico Ortopedico Traumatologico Gaetano Pini-CTO, Milan, Italy; c Scuola Di Specializzazione in Ortopedia e Traumatologia Università Degli Studi Di Milano, Milan, Italy; d U.O.C. Radiodiagnostica, Azienda Socio Sanitaria Territoriale Centro Specialistico Ortopedico Traumatologico Gaetano Pini-CTO, Milan, Italy; e Laboratory of Applied Biomechanics, Department of Biomedical Sciences for Health, Università Degli Studi Di Milano, Milan, Italy; f U.O.C. 1° Clinica Ortopedica, ASST Centro Specialistico Ortopedico Traumatologico Gaetano Pini-CTO, Milan, Italy; g Research Center for Adult and Pediatric Rheumatic Diseases (RECAP-RD), Department of Biomedical Sciences for Health, Università Degli Studi Di Milano, Milan, Italy

**Keywords:** Recalcitrant lateral elbow pain, Elbow instability, HELP US test, Varus stress, Diagnostic evaluation, Epicondylitis

## Abstract

**Background:**

Recent evidence indicates that lateral elbow pain may not stem solely from extra-articular or tendon-related sources, but rather be part of a multifactorial process that also involves intra-articular factors. Therefore, diagnosis of minor elbow instability is often delayed, waiting for conservative measures to assess the problem. A timely diagnosis could help achieve better patient care. While ultrasound (US) stress tests have been instrumental in evaluating joint instability across various anatomical sites, their role in diagnosing minor instability of the lateral elbow remains unexplored. The aim of this study is to assess the presence of lateral joint opening in a functional position using dynamic varus-stressed US in patients with clinically suspected atraumatic minor instability.

**Materials and methods:**

Patients with suspected minor instability were compared to a control group with nonpathologic elbows. Eligible patients underwent varus stress US of the elbow with 70° of flexion of the elbow and a 3 kg weight fixed to the wrist. The presence of lateral widening and its increase were documented and compared between the groups. The test was named Highlight Elbow Lateral Pathology with UltraSound (HELP US). Sixty-five elbows were evaluated in this study. There were 35 patients in the case group (80% male, mean age 47 years) and 30 healthy controls (77% male, mean age 49 years).

**Results:**

The mean lateral elbow joint width was 26.7% in the pathological group, meanwhile, in the control arm, the mean lateral elbow joint width increase was 3.2%. The groups had a significant difference in the widening of the lateral elbow during the HELP US test (*P < .01*). Within the cases, a total of 17 patients (48%) had a history of previous corticosteroid injections. The mean lateral elbow joint width increase was 27%, showing no difference with those patients who showed no history of previous corticosteroid injections.

**Conclusions:**

The HELP US test can detect changes in the lateral elbow compartment and can depict lateral elbow articular space widening. This is a valid diagnostic tool and should be implemented in evaluating all patients complaining of lateral elbow pain. The HELP US test could help physicians screen for a timely diagnosis of minor instability and speed up the request for second-level imaging studies to those patients that effectively require it the most.

Degeneration and tendinosis of the common extensor origin, specifically the extensor carpi radialis brevis, are generally considered the main causes of lateral epicondylitis (LE) or tennis elbow.^15^ However, recent evidence suggests that the extra-articular or tendon-related source may not be the sole cause of lateral elbow pain but rather part of a multifactorial process involving extra-articular, intra-articular, and systemic factors.^8^ Arthroscopy is the gold standard for identifying intra-articular findings, with flexion arthro-computed tomography as a close alternative.

Chronic lateral elbow pain has been described in the setting of atraumatic posterolateral rotator instability (PLRI),^12^,^19^ repeated corticosteroid injections,^5^ and symptomatic minor instability of the lateral elbow (SMILE).^1^ Clinical and radiological diagnosis of these conditions is often troublesome, since, other than recalcitrant pain, no specific test is available for their precise diagnosis. Often first-line imaging studies are not helpful. A 12-month wait-and-see policy is recommended as the standard of care in chronic lateral elbow pain.^13^ A timelier and more cost-effective diagnostic tool that can identify mechanical sources of pain would be highly beneficial and could expedite a comprehensive patient assessment.

Unfortunately, clinical diagnostic tools to evaluate the presence of patholaxity or minor forms of instability are lacking and limited to pilot studies and technical descriptions.^1^^,^^18^

While ultrasound (US) stress tests have been instrumental in evaluating joint instability across various anatomical sites (knee for example), their role in diagnosing minor instability of the lateral elbow remains unexplored.^14^ In comparison to stress radiographs, this method avoids radiation, allows for dynamic assessment, and enables side-to-side comparison.^16^

This study aims to evaluate whether there is lateral joint opening in a functional position with dynamic varus-stressed US (US instability) in cases of clinical suspect of atraumatic minor instability when compared to healthy controls. The analysis allows us to Highlight Elbow Lateral Pathology through UltraSound, which was named the Highlight Elbow Lateral Pathology with UltraSound (HELP US) test.

The hypothesis is that patients suspected of minor instability of the lateral elbow present abnormal widening of the lateral elbow during the HELP US test.^17^

## Materials and methods

### Inclusion and exclusion criteria

The design is a comparative cohort study of a retrospectively enrolled group of patients vs. a control group, with level of evidence III.

Patients were included in the study if they met the following criteria: 1) at least 4 months of lateral elbow pain, 2) inconclusive results from initial diagnostic imaging (eg, X-ray), 3) suspicion of minor instability (SMILE, atraumatic PLRI, or subtle PLRI without significant trauma), and 4) age 18 or older. Cases were excluded in case of previous infection, deformity, or traumatic instability. Patients were suspected of SMILE if they presented positive supination and antero-lateral pain test and/or posterior elbow pain by palpation-extension of the radiocapitellar joint tests and if direct anterior or posterior palpation of the radial head was more painful than palpation at the level of the lateral epicondyle.^3^ Atraumatic PLRI was suspected in patients affected by lateral elbow pain, especially provoked by leaning on the hand in a slight flexion and forearm in supination. All consecutive patients were referred to our institution between August 2021 and February 2024. Subjects without a history of elbow trauma or other diagnosed pathologies were included as healthy controls. Subgroup analysis was performed in the pathological group and the corticosteroid injections received before the tests were recorded.

Institutional approval of the study protocol was obtained before the study began (resolution n° 138 dated 18 March 2021- 233_2021) and was conducted according to the Declaration of Helsinki.

### HELP US test

Stressed US provides functional imaging and detailed findings of articular derangement while the patient is not exposed to ionizing radiation.^7^

HELP US was performed using a linear 4-15 MHz transducer US machine by the same musculoskeletal-trained senior radiologist, in both cases and controls. A standardized protocol was applied focusing on the lateral elbow joint during provocative maneuvers.

**Figure 1:**
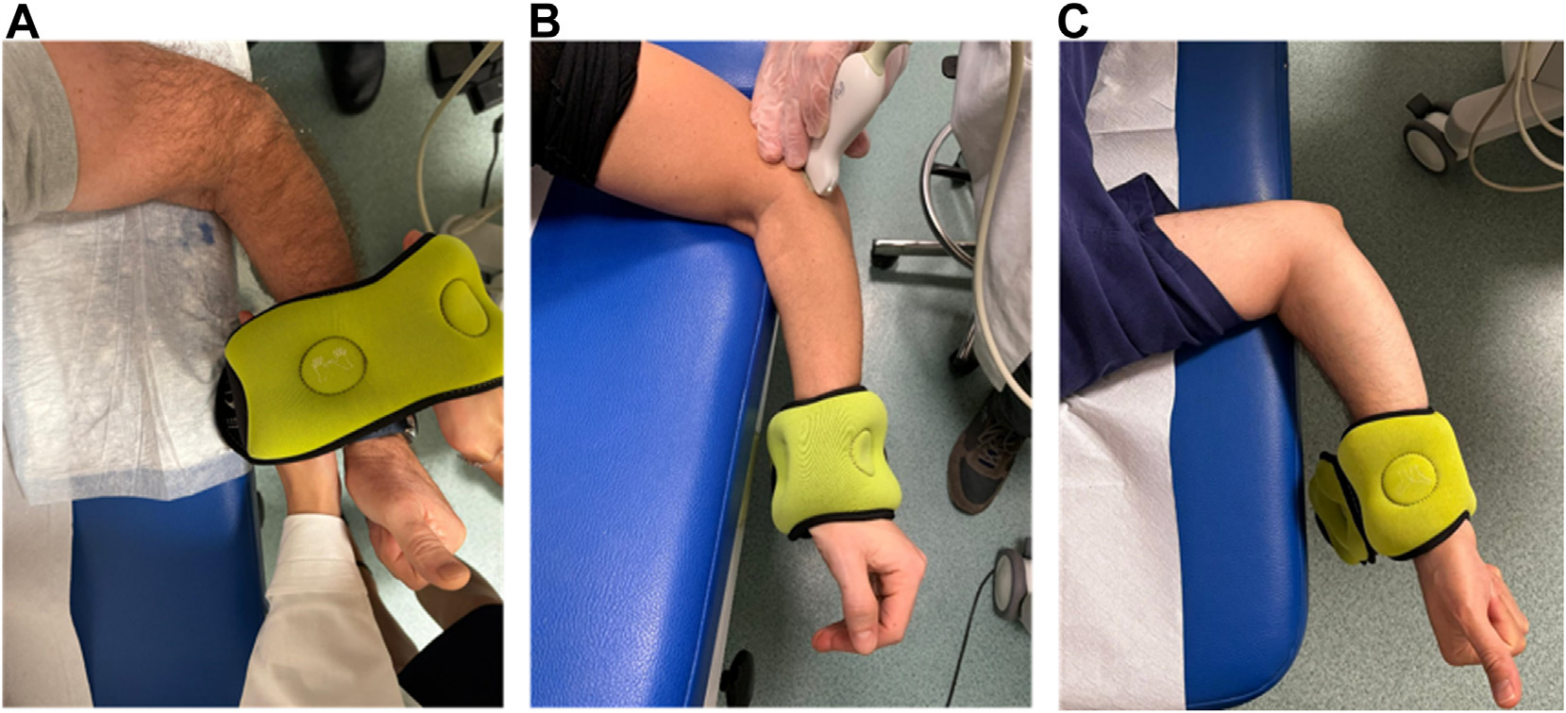
Patient position prone on the scanning table with the arm abducted at 90°, the forearm in a suspended position with thumb up (**A**, starting position), while the elbow is flexed at 70° (**B**, US scan assessment) with 3 kg weight on patient’s wrist. The thumb is extended to control the orientation of the arm to limit the bias of humeral rotation under stress and to control that no rotation in the shoulder can create a bias in the elbow assessment (**C**).

The patient is positioned prone on the scanning table, with the arm abducted at 90° and the forearm in a suspended position, while the elbow is flexed at 70° to exclude the involvement of bony stabilizers (Fig. 1). The thumb is extended to control the orientation of the arm and to limit the bias of humeral rotation under stress. The US scan is then positioned on a standard longitudinal plane visualizing the lateral articular space while keeping the lateral epicondyle contour as proximal reference and the proximal shaft of the radius as distal reference. A weight of 3 kg positioned on the patient’s wrist is then applied while ensuring a 70° angle of elbow flexion is maintained (Fig. 1).

**Figure 2:**
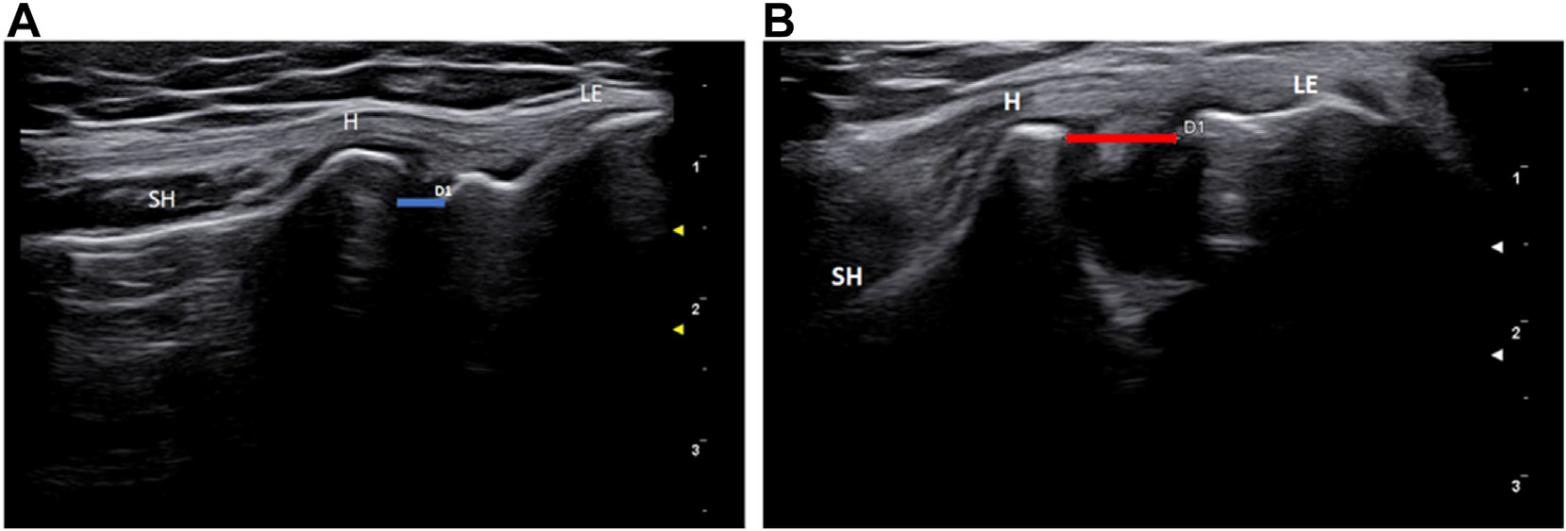
(**A**) Assessment of the lateral articular space in a baseline condition (*blue line*, D1). (**B**) Measurement of the lateral articular space during stress test (*red line*, D1). *LE*, lateral epicondyle; *H*, humeral head; *SH*, radial shaft.

The US measurement of the articular lateral space is obtained by positioning two calipers, respectively. on the radial and humeral articular surface at rest and during stress maneuvers, after the 3 kg weight has been applied, to assess for any significant widening of the lateral elbow joint (Fig. 2). The measure is expressed in millimeters (mm) between the radial head and humerus. The difference between the baseline and stressed condition was calculated. Differences of less than 10% were recorded as 0% due to the inherent difficulty in accuracy during assessment. Conversely, differences of more than 100% were grouped within the 100% widening group.

### Statistical analysis

Demographic data, as well as data regarding the diagnosis and treatment of elbow pathology and information on US findings, were entered into a spreadsheet for analysis and stored in a digitalized and centralized database compliant with the European Union’s General Data Protection Regulation for analysis.

Distributions were assessed for normality or log normality using the Shapiro–Wilk test. Unpaired t-tests were conducted for every group of measurements, with a significance level set at *P <* .05. After the collection of painful patients (cases), a desired power of 80% and a significance level of 5% (two-sided comparison of proportions) were chosen, and a total sample size of 65 measurements was considered sufficient to test our hypothesis. An adequate control group was recruited according to this analysis.

## Results

Sixty-five participants were enrolled in the study: 35 patients in the case group (80% male, mean age 47 years) and 30 healthy controls (77% male, mean age 49 years). None of the included subjects was subsequently excluded, no difficulties emerged in performing US measurements, and the capitellar and radial articular surfaces were always easily identifiable. The groups were comparable in age and gender (*P > .05*).

The mean radio-capitellar distance in the cases at baseline condition was 3.7 mm, compared to 3.2 mm in the control population (*P* = .01). After the stress test, the distance was 4.4 mm in cases and 3.3 mm in controls (*P* < .01). The mean lateral elbow joint width increase expressed as a percentage (%) between baseline and stressed position was 26.7% (range 0%-100%; SD, 26) in the index group vs. 3.2%% (range 0%-25%°; SD, 6.3) of the controls. Cases exhibited a significantly higher lateral joint width under varus stress US provocative maneuvers compared to the control group (*P ≤ .1*). Healthy subjects did not display an increase in widening between baseline and stressed US (*P = .11*). The results of US measurements are summarized in Table 1. These findings suggest that widening is a prominent feature in cases of patients with suspect of minor instability. A subgroup analysis was performed in the case population, concerning the incidence of corticosteroid injections and the possible influence that injections could have on the outcome. A total of 17 patients (48%) had a history of previous corticosteroid injections with an average of 3.25 injections per patient. The mean lateral elbow joint width difference was 27%, the same as the one registered with a population that had no history of previous corticosteroid injections.

**Table I:** Minor instability cases patients and healthy controls characteristics and width means.

	Patients (n = 35)	Healthy controls (n = 30)	*P* value
Mean age	47	49	*P* > .5
Demographics (M%)	80%	77%	*P* > .5
Mean width delta % ± SD	27% ± 1%	9% ± 0.5%	*P* = .1
Min width delta %	0%	0%	
Max width delta %	100%	25%	
Baseline, mean (mm)	3.7 mm	3.2 mm	*P* = .1
Stress test, mean (mm)	4.4 mm	3.3 mm	*P* < .1

*SD*, standard deviation.

## Discussion

The main result of this study is that patients with recalcitrant lateral elbow pain exhibited a significantly higher lateral joint width under the HELP US test compared to the control group. This finding confirms objective joint lateral opening.^11^ A difference has been found also at baseline conditions as well before stress testing. On the other hand, corticosteroid injections do not appear to play a role in lateral joint widening.

**Figure 3:**
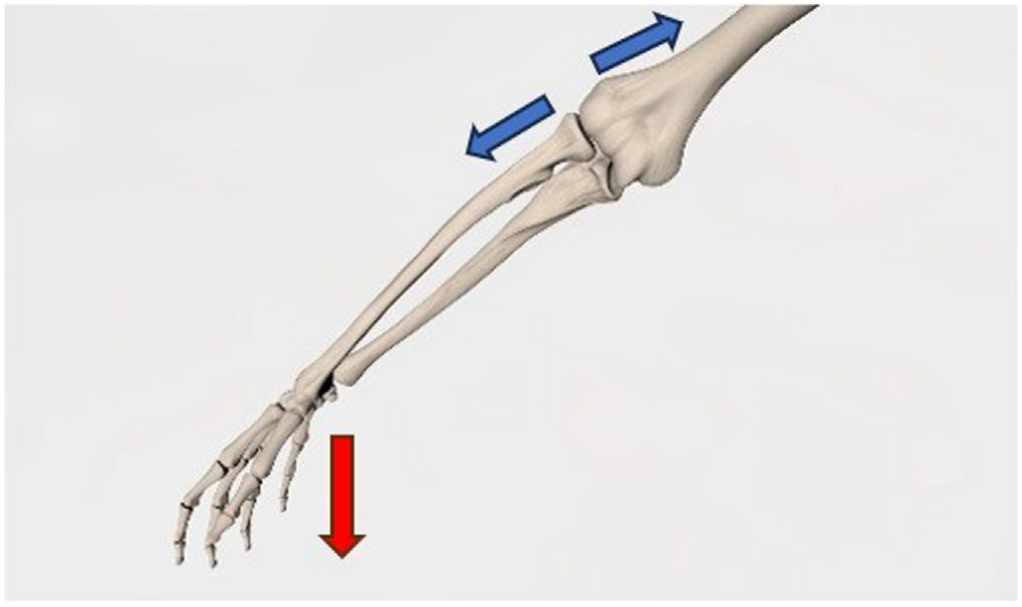
Anatomical representation of the US stress test. The weight on the wrist emphasizes the joint widening in pathological conditions, whereby the extensor muscles are unable to provide sufficient resistance to the applied load. *US*, ultrasound.

Joint widening (as the elbow) has been considered a cardinal sign of instability, prompting further investigation and intervention.^9^ The position is chosen as it better represents the standard flexion during daily desk activities, and it mainly represents the angle of flexion and varus stress the elbow is exposed to when holding objects.^20^,^21^ Cadaveric studies have shown how the radial component of the lateral collateral ligament affects the stability of the elbow, especially at 70° degrees of flexion and varus load application, in the SMILE population. For this reason, the US stress test was performed in the aforementioned position, with the arm abducted at 90° and the forearm at 70° of flexion (Fig. 3). The ease with which US measurements were performed and the consistent identification of the capitellar and radial articular surfaces highlight the intra observer reliability and validity of the obtained data.

Lateral elbow pain is commonly associated with LE. In fact, patients suffering from persistent lateral elbow pain often wait more than 12 months before seeking second-level imaging, as most cases of LE resolve with conservative treatment within this period. However, the early identification of minor instability can accelerate the diagnostic process for some patients and enhance their overall care. This proactive approach may reduce the necessity for related interventions, such as bracing, physical therapy, and injections of corticosteroids, collagen, or PRP, while also minimizing intra-articular complications linked to minor elbow instability, ultimately leading to a better prognosis. US can aid in diagnosing lateral ligamentous laxity, thereby confirming clinical suspicions.

Our study confirms that joint widening is present in subjects of atraumatic minor elbow instability. The observed widening of the lateral elbow joint suggests a pathophysiological mechanism underlying these injuries. With the forearm hanging in the void, the extensor muscles are needed and sufficient to counteract the action of gravity, especially when clinically evaluating the patients. When an exceeding weight is applied, the extensor muscles are not sufficient to contrast this excessive load in pathological conditions and joint widening is more likely to occur. This study highlights how not only during stress conditions but also at baseline, there is a difference between healthy and instable patients. This could lead to the gravity being enough to show at US patholaxity of the joint, which is then further confirmed by stress testing.

Arthroscopic evaluation of SMILE patients has shown the presence of horizontal instability, in line with radial component of the lateral collateral ligament laxity, rather than vertical instability, which is more likely found in the setting of PLRI and the ulnar band of lateral collateral ligament (LCL) laxity and lesion.^2^ On the other hand, the ulnar band of LCL seems to be involved in the setting of atraumatic PLRI, so much so that arthroscopic plication of the ulnar band of LCL has been proposed as treatment in this setting of patients.^4^ One plausible explanation for lateral widening observed in our study could be the involvement of combined patholaxity mechanisms in symptom generation. These subtle yet intricate mechanisms may contribute to symptoms without necessarily manifesting physical joint instability, as evidenced by the absence of laxity during physical examination, negative drawer sign, and valgus stress test, but with widening on US examination. Forearm rotation in particular in supination as done in this test could influence our findings, leading to a higher increase in joint widening. To avoid this bias, thumb positioning was carefully visualized throughout US examination and the risk of rotational bias was similar in both cases and controls. On the other hand, internal rotation of the humerus, which could also occur, would decrease the joint widening. Therefore, it would not compromise our results, which, regardless, show a widening of the joint line.

Subtle ligamentous laxity, proprioceptive deficits, and dynamic muscle imbalance may contribute to symptom generation in patients who display minor instabilities.^1^ US assessment of the posterolateral elbow ulno-humeral gap with and without posterolateral drawer testing^6^ showed differences in the lateral ulno-humeral gap between stressed and resting conditions in healthy individuals. Further studies to compare these results with assessments in PLRI and SMILE patients could give a better insight into the different pathophysiological mechanisms underlying these conditions.

The HELP US test may be implemented as a diagnostic tool, driving treatment strategies for patients in which a minor instability of the elbow is suspected. Anyhow, the findings emphasize the multifactorial nature of lateral elbow instability, which warrants a comprehensive diagnostic approach that extends beyond stress US examination.

Multiple corticosteroid injections to the lateral elbow have been showed to be a risk factor for atraumatic PLRI.^10^ In our study, we did not find any correlation, and the widening of the lateral elbow joint was not greater in patients with a history of corticosteroid injections. Future studies involving a larger patient population should explore the role of corticosteroid injections in the pathology and treatment of lateral elbow pain.

**Figure 4:**
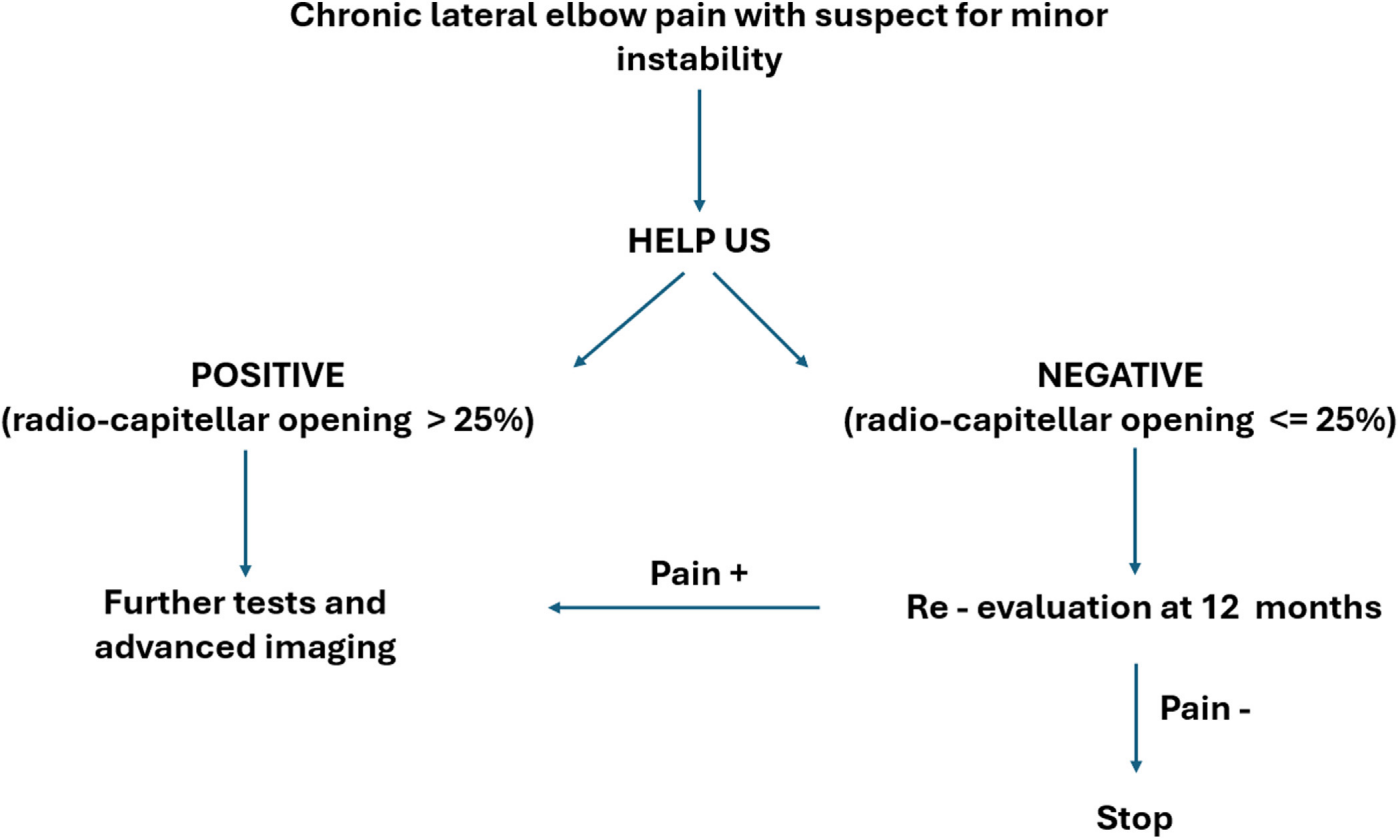
Flowchart for the use of the HELP US stress test in clinical practice. *US*, ultrasound; *HELP US*, Highlight Elbow Lateral Pathology with UltraSound.

This study highlights the utility of US measurements in assessing joint instability, particularly in the context of minor instabilities of the elbow. We propose to use the HELP US test as a cornerstone in the diagnostic algorithm for these difficult patients (Table 1). Based on the data collected from our study and the wide range of measurements observed in patients with a strong suspicion of atraumatic instability, in contrast to the limited range found in the control group, we suggest using 25% of lateral joint opening (which corresponds to the maximum value observed in our control population) as a cut-off. Patients falling below this threshold should be further evaluated with more invasive tests, such as magnetic resonance imaging, Arthro-computed tomography, or Arthro- magnetic resonance imaging) to avoid overlooking cases with a negative HELP US stress test. Additionally, more advanced imaging^22^ should be considered only when pain persists for over 12 months despite conservative treatment (Fig. 4). Using this cutoff and based on our data, the test we propose demonstrates a sensitivity of 40% and a specificity of 100%. Consequently, it enables us to classify positive patients as cases, effectively identifying true negatives.

Indeed, US offers a noninvasive, cost-effective means of evaluating joint morphology and function, providing valuable information for clinical decision-making. Integrating US assessment into the diagnostic workup of these patients may enhance diagnostic accuracy and facilitate timely intervention, thereby improving patient care and prognosis (Fig. 4).

It is important to recognize the limitations of this study, including its relatively small sample size and potential confounding factors, such as individual muscle strength and hyperlaxity, which may have affected the results despite the two populations being comparable in terms of age and gender. Additionally, US does not enable surgeons to differentiate between various types of atraumatic instability; this distinction can currently only be made through arthroscopy. US measurements were conducted by a single expert radiologist, so further studies should evaluate the reproducibility of this technique. To mitigate biases related to absolute measurements calculated to the tenth of a millimeter, we obtained the difference in widening percentage (Delta%) to generalize the results. Future research should aim to address these limitations and validate the current findings with larger, more comprehensive studies. Moreover, additional investigations should compare minor and major instability pathologies using US evaluation.

## Conclusion

The HELP US test showed a notable widening of the radio-capitellar joint in instances of confirmed lateral elbow pain with a suspicion of minor instability. These findings support established beliefs about the effectiveness of stress tests in detecting joint instability. Corticosteroid injections, however, do not appear to be associated with progressive widening of the elbow joint in cases of minor instability; further research is needed to confirm this. The HELP US test could help physicians achieve a timely diagnosis of minor instability by speeding up the request for second-level imaging studies to those patients who require it the most.

## Disclaimers:

Funding: No specific funding was received from any public, commercial, or not-for-profit bodies to carry out the work described in this article.

Conflicts of interest: Aurelien TRAVERSO received financial support for his fellowship from Bonebridge and from SICPA. The other authors, their immediate families, and any research foundation with which they are affiliated have not received any financial payments or other benefits from any commercial entity related to the subject of this article.
